# Predictors of response to anti-CGRP monoclonal antibodies: a 24-week, multicenter, prospective study on 864 migraine patients

**DOI:** 10.1186/s10194-022-01498-6

**Published:** 2022-11-01

**Authors:** Piero Barbanti, Gabriella Egeo, Cinzia Aurilia, Claudia Altamura, Florindo d’Onofrio, Cinzia Finocchi, Maria Albanese, Marco Aguggia, Renata Rao, Maurizio Zucco, Fabio Frediani, Massimo Filippi, Roberta Messina, Sabina Cevoli, Antonio Carnevale, Giulia Fiorentini, Stefano Messina, Francesco Bono, Paola Torelli, Stefania Proietti, Stefano Bonassi, Fabrizio Vernieri, for the Italian Migraine Registry study group

**Affiliations:** 1 Headache and Pain Unit, IRCCS San Raffaele Roma, Via della Pisana 235, 00163 Rome, Italy; 2 San Raffaele University, Rome, Italy; 3 Headache and Neurosonology Unit, Headache and Neurosonology Unit, Fondazione Policlinico Campus Bio-Medico, Rome, Italy; 4 Neurology Unit, San Giuseppe Moscati Hospital, Avellino, Italy; 5 IRCCS Ospedale Policlinico San Martino, Genoa, Italy; 6 Regional Referral Headache Center, Neurology Unit, University Hospital Tor Vergata, Rome, Italy; 7 Neurology and Stroke Unit, Asti Hospital, Asti, Italy; 8 Departement of Neurological Sciences and of Vision, P.le Spedali Civili, Brescia, Italy; 9 Headache Center, Neurology Unit, San Camillo-Forlanini Hospital, Rome, Italy; 10 Headache Center, ASST Santi Paolo Carlo, Milan, Italy; 11 Neurology Unit, Neurorehabilitation Unit, Neurophysiology Unit, Headache Center, Vita-Salute San Raffaele University and San Raffaele Scientific Institute, Milan, Italy; 12 IRCCS Istituto delle Scienze Neurologiche di Bologna, Bologna, Italy; 13 Headache Center, Neurology Unit, San Filippo Neri Hospital, Rome, Italy; 14 Department of Neurology-Stroke Unit, Laboratory of Neuroscience, Istituto Auxologico Italiano, IRCCS, Milan, Italy; 15 Center for Headache and Intracranial Pressure Disorders, Neurology Unit, A.O.U. Mater Domini, Catanzaro, Italy; 16 Unit of Neurology, Department of Medicine and Surgery, Headache Center, University of Parma, Parma, Italy; 17 Clinical and Molecular Epidemiology, IRCCS San Raffaele Roma, Rome, Italy

**Keywords:** Migraine, Predictors, AntiCGRP mAbs, Unilateral cranial autonomic symptoms, Allodynia, Registry

## Abstract

**Background and objectives:**

The identification of predictors of response to antiCGRP mAbs could favor tailored therapies and personalized treatment plans. This study is aimed at investigating predictors of ≥ 50%, ≥ 75% and 100% response at 24 weeks in patients with high-frequency episodic (HFEM: 8–14 days/month) or chronic migraine (CM).

**Methods:**

This is a large, multicenter, cohort, real-life study. We considered all consecutive adult patients affected by HFEM or CM who were prescribed antiCGRP mAbs for ≥ 24 weeks in 20 headache centers. Patients were interviewed face-to-face using a shared semi-structured questionnaire carefully exploring socio-demographic and clinical characteristics. Patients received subcutaneous erenumab (70 mg or140 mg, monthly), galcanezumab (120 mg monthly, following a 240 mg loading dose), or fremanezumab (225 mg, monthly or 675 mg, quarterly) according to drug market availability, physician’s choice, or patient’s preference. The primary endpoint of the study was the assessment of ≥ 50% response predictors at 24 weeks. Secondary endpoints included ≥ 75% and 100% response predictors at 24 weeks.

**Results:**

Eight hundred sixty-four migraine patients had been treated with antiCGRP mAbs for ≥ 24 weeks (erenumab: 639 pts; galcanezumab: 173 pts; fremanezumab: 55 pts). The ≥50% response (primary endpoint) in HFEM was positively associated with unilateral pain (UP) + unilateral cranial autonomic symptoms (UAs) (OR:4.23, 95%CI:1.57–11.4; *p* = 0.004), while in CM was positively associated with UAs (OR:1.49, 95%CI:1.05–2.11; *p* = 0.026), UP + UAs (OR:1.90, 95%CI:1.15–3.16; *p* = 0.012), UP + allodynia (OR:1.71, 95%CI:1.04–2.83; *p* = 0.034), and negatively associated with obesity (OR:0.21, 95%CI:0.07–0.64; *p* = 0.006). The 75% response (secondary endpoint) was positively associated with UP + UAs in HFEM (OR:3.44, 95%CI:1.42–8.31; *p* = 0.006) and with UP + UAs (OR:1.78, 95%CI:1.14–2.80; *p* = 0.012) and UP + allodynia (OR:1.92, 95%CI:1.22–3.06; *p* = 0.005) in CM. No predictor of 100% response emerged in patients with HFEM or CM.

**Conclusions:**

A critical evaluation of headache characteristics indicating peripheral or central sensitization may help in predicting responsiveness to antiCGRP mAbs in HFEM and CM. A more precise pain profiling may represent a steppingstone for a mechanism-based approach and personalized treatment of migraine with compounds targeting specific molecular mechanisms.

## Introduction

Monoclonal antibodies (mAbs) targeting the Calcitonin Gene Related Peptide (CGRP) or its receptor have been launched since 2018 for the prevention of episodic and chronic migraine and represent the first specific and selective migraine prophylactic treatment [1]. Despite some differences in terms of type (fully human, humanized), target (CGRP, CGRP receptor), way of administration (subcutaneous, intravenous) and dosing (monthly, quarterly), antiCGRP mAbs share a remarkably similar clinical profile, being effective and well tolerated in patients with episodic or chronic migraine, even in presence of medication overuse or prior therapeutic failures (i.e. treated unsuccessfully - in terms of either efficacy or tolerability, or both - with 2 to 4 preventive treatments) [2]. Their distinguishing feature is the considerable proportions of responders (≥ 50% reduction in monthly migraine days) and super-responders (≥ 75% reduction in monthly migraine days), and the excellent efficacy and tolerability ratio which represents a substantial step forward compared to the usual standard of care [3, 4]. Thus, mAbs to CGRP emerge as a tremendous opportunity for alleviating migraine and lifting patients’ burden, ultimately improving their quality of life [5].

Yet it should be recognized that some clinical and economic concerns exist, because antiCGRP mAbs are ineffective in one third of the patients and their elevated cost has led some European Countries to apply restrictive reimbursement norms. In this view, the identification of response predictors could have a clinical and economic impact, being of help in implementing tailored therapies and personalized treatment plans, optimizing resource allocation [6].

Some studies have suggested that responsiveness to antiCGRP mAbs could be related to several demographic and clinical features - including age, sex, body mass index, basal migraine frequency and disability, pain side and severity, allodynia, dopaminergic symptoms, response to triptans and psychiatric comorbidities and personality trait. The heterogeneity of these findings could depend on differences on populations studied, sample sizes, study designs and clinical endpoints investigated [7–18].

Seeking reliable information that might shed light on socio-demographic or clinical profiling of responders to antiCGRP mAbs, we designed this study aimed at investigating potential predictors of response (≥ 50% response rate) or super-response (≥ 75%, 100% response rates) at 24 weeks in patients affected by high-frequency episodic (HFEM: 8–14 days/month) or chronic migraine (CM) in a large, prospective, multicenter, real-life population.

## Methods

### Trial design and participants

This is a multicenter, cohort, real-life study ongoing at 20 headache centers distributed throughout 7 Italian regions from December 20th, 2018, with the latest patient recruited on March 7th, 2021. This study is part of the I-NEED (Italian NEw migrainE Drugs database) project, included in the Italian Migraine Registry (I-GRAINE). All consecutive ≥ 18 years old patients affected by HFEM or CM [19] who were prescribed antiCGRP mAbs for ≥ 24 weeks according to the criteria required by the Italian Medicine Agency (AIFA) (*adult patients with* *≥ 8 monthly migraine days over the last 3 months, MIDAS score > 11, and documented failure, contraindications, or low tolerability to > 3 pharmacological classes of migraine preventive medications among beta-blocker, anticonvulsants and tryciclics, or onabotulinum toxinA for CM*) were evaluated [20].

The study was approved by the IRCCS San Raffaele Roma Institutional Review Board as coordinating center (11/2018) and mutually recognized by the other local Institutional Review Boards. Each participant provided informed consent. After signing the informed consent, all patients underwent a thorough physical and neurological evaluation and were interviewed by specifically trained, board-certified neurologists with face-to-face interviews using a shared semi-structured questionnaire carefully exploring the following socio-demographic and clinical characteristics [21]: sex, age, body mass index (BMI) and BMI classes (underweight: < 18.5; normal weight: 18.5 to < 25; overweight: 25 to < 30; obesity: ≥ 30), disease duration, migraine type, migraine frequency at baseline, pain side (unilateral pain = hemicranial location, same side or alternating side), quality and intensity [using the Numerical Rating Scale (NRS) score], disability [using the Headache Impact Test-6 (HIT-6) score], presence of unilateral cranial autonomic symptoms (defined as ≥ 1 of the following unilateral symptoms during the migraine attack: lacrimation, eye redness, nasal congestion, ptosis, eyelid swelling, miosis or forehead/facial sweating) [21], allodynia, presence of dopaminergic symptoms (defined as ≥ 1 of the following symptoms during prodromes, headache stage or postdromes: yawning, somnolence, nausea, vomiting, mood changes, fatigue or diuresis) [22], response to triptans, response to onabotulinum toxinA; concomitant prophylaxis; prior treatment failures; comorbidities and concomitant medications [21]. The current study, as part of the Italian Migraine Registry initiative included a large proportion of patients admitted to the 20 participating centers in the study period. Given the large number of subjects recruited, the sample size of the study, i.e., 864 patients (208 HFEM and 565 CM), was determined by a non-probability convenience sampling. The size of the convenient sample is considerably larger than that needed to test a single hypothesis, nevertheless the observational nature of the study does not imply ethical concern, and a substantially larger sample size is recommended when the study involves the testing of many hypotheses [23]. According to the findings of our previous study [21], symptoms related to peripheral sensitization (unilateral pain: UP; unilateral cranial autonomic symptoms: UAs) or central sensitization (allodynia) were explored also in combination in each subject as follows: UP associated with UAs (UP + UAs), UP associated with allodynia (UP + allodynia); UP associated with UAs and allodynia (UP + UAs + allodynia).

All the patients were antiCGRP mAbs naïve. Starting 28-days prior to the first antiCGRP mAb dose, and throughout the whole study duration, patients filled-out a paper-pencil diary recording monthly migraine days (for HFEM), monthly headache days (for CM), monthly acute medication intake, pain intensity of the monthly most painful attack, and pain disability. Patients received subcutaneous erenumab (70 mg or 140 mg) every 28 days, galcanezumab (120 mg following a loading dose of 240 mg) every 30 days, or subcutaneous fremanezumab 225 mg monthly (every 30 days) or 675 mg quarterly (every 90 days) according to drug market availability, physician’s choice, or patient’s preference. In agreement with the Italian distribution rules, the pharmacy provides 3 mAbs doses to each patient at week 0, 3 doses at week 12 and 6 doses at week 24.

Concomitant migraine prophylaxis was allowed. Patients were re-evaluated at 12 weeks and 24 weeks, as required by AIFA regulation.

The primary endpoint of the study was the assessment of ≥ 50% response predictors at 24 weeks. Secondary endpoints included ≥ 75% and 100% response predictors at 24 weeks.

### Statistical analysis

Categorical data, were analysed with the χ2 test or Fisher’s exact test when appropriate. Shapiro–Wilk test was used for normality determination of the data. Student’s t-test or one-way ANOVA were used to compare normal distributed data, and the Mann-Whitney U-test was used for non-normal distributed data. The proportion of missing data was low, in most cases below 5%. The highest proportion of missing data was found for BMI, i.e., 133 patients (15.4%). A sensitivity analysis compared selected features of subjects included in the study versus missing subjects to rule out the hypothesis of a selection bias. Whenever the proportion of missing was higher than 5%, monthly migraine days, the M/F ratio, and the mean age of the two groups were compared. In no cases significant differences were found. Binary logistic regression was used for the multivariate analysis. All models investigated the associations between > 50%, > 75%, and 100% response and potential clinical and epidemiological predictors. Variables significantly associated with response in the univariate analysis (including their combinations), variables associated with migraine characteristics in the literature or in our previous studies [21], and gender and age as fixed parameters were included as covariates in logistic regression models. A backward removal procedure of all the independent variables that did not substantially contribute to the regression equation (*p* <  0.10) was applied. The large number of hypotheses tested reveals the substantially explorative nature of the analysis. *P*-values generated by univariate and multivariate testing have a two-fold meaning, to generate hypotheses which will address further research on this topic, and to rank the credibility of study findings [24]. As an additional data, the Holm-Bonferroni method was applied to the analysis of potential response predictors to deal with the effect of multiple hypothesis testing. The presence of multiple co-primary outcomes of clinical interest, suggested to combine these into a composite outcome, as already done in our previous research [21]. The three variables reflecting different features of central/peripheral sensitization were evaluated jointly or two-by-two in the univariate analysis and fitting different regression models [25]. To check the assumption that different components of the combined outcome share similar influence on the probability of response, the ORs of individual components were estimated and reported together with estimates for combined measures [26]. The results of multivariate analysis are extensively reported only for 50% and 75% response because of the small number of patients with a 100% response. All models were compared with the Akaike information criterion (AIC), while model calibration and discrimination were evaluated with the Hosmer-Lemeshow goodness-of-fit test. The analysis was performed using SPSS software package *(v27.0)*, and R Statistical Software *(v3.6.2)*.

## Results

At the time of the analysis, 864 migraine patients had been treated with antiCGRP mAbs for ≥ 24 weeks (erenumab: 639 pts; galcanezumab: 173 pts; fremanezumab: 55 pts). Their demographic and clinical characteristics are reported in Table 1. Patients were mostly females (78.1%), affected by CM (75.9%), with concomitant medication overuse headache (87%) and were characterized by very high disability (HIT-6 score: 66.0 ± 9.2) and multiple prior therapeutic failures (4.9 ± 2.3). Patients affected by CM differed from those with HFEM for higher prevalence of obesity (5.7% vs 0.6%; *p* = 0.032), NRS score (7.8 ± 1.3 vs 7.5 ± 1.4; *p* = 0.005), pain side (unilateral 46.8% vs 58.2%; p = 0.005), UAs (51.4% vs 39.8%; *p* = 0.004), allodynia (59.9% vs 44.2%; *p* <  0.001), prior therapeutic failures (5.2 ± 2.3 vs 4.1 ± 2.2; *p* < 0.001), response to triptans (61.7% vs 70.2%; *p* = 0.036), response to onabotulinum toxin (7.5% vs 23.1%: *p* < 0.001) and psychiatric comorbidities (22.6% vs 13.9%; *p* = 0.007).

**Table 1:** Demographic and clinical features of migraine patients by high-frequency episodic (HFEM) or chronic migraine (CM)

	Number (%) or mean ± SD			***P***-value
	All	HFEM	CM	
**Patients**	864	208 (24.1)	656 (75.9)	
**Age,** yrs	47.8 ± 11.5	48.2 ± 11.0	47.7 ± 11.6	0.629
**Females**	675 (78.1)	158 (76.0)	517 (78.8)	0.388
**BMI**	23.2 ± 3.7	22.7 ± 2.7	23.3 ± 3.9	0.069
**BMI class**				**0.032**
*Underweight*	42 (5.8)	8 (4.6)	34 (6.1)	
*Normal*	504 (68.9)	126 (73.3)	378 (67.6)	
*Overweigh*	152 (20.8)	37 (21.5)	115 (20.6)	
*Obesity*	33 (4.5)	1 (0.6)	32 (5.7)	
**Disease duration*,*** yrs	30.3 ± 12.6	29.5 ± 12.3	30.6 ± 12.7	0.305
**MMDs/MHDs at baseline**	20.6 ± 7.5	10.9 ± 2.0	23.7 ± 5.8	–
**MOH**		–	571 (87.0)	–
**MOH duration**		–	9.1 ± 8.9	–
**Monthly analgesic intake at baseline**	23.8 ± 21.2	12.6 ± 5.5	27.4 ± 23.0	**< 0.001**
**NRS score**	7.7 ± 1.3	7.5 ± 1.4	7.8 ± 1.3	**0.005**
**UP**	418 (49.5)	117 (58.2)	301 (46.8)	**0.005**
**Pain quality**				0.286
*Pulsating*	556 (67.2)	127 (64.8)	429 (68.0)	
*Pressing/tightening*	243 (29.4)	59 (30.1)	184 (29.2)	
*Other*	28 (3.4)	10 (5.1)	18 (2.8)	
**UAs**	406 (48.6)	80 (39.8)	326 (51.4)	**0.004**
**Allodynia**	472 (56.2)	89 (44.2)	383 (59.9)	**< 0.001**
**Dopaminergic symptoms**	563 (67.5)	146 (72.6)	417 (65.9)	0.075
**UP + allodynia**	261 (58.0)	55 (53.9)	206 (59.1)	0.343
**UP + UAs**	248 (55.4)	54 (53.5)	194 (55.9)	0.664
**UP + UAs + allodynia**	221 (64.6)	47 (59.5)	174 (66.2)	0.277
**Triptan responders**	512 (63.8)	139 (70.2)	373 (61.7)	**0.036**
**Concomitant prophylaxis**	464 (56.0)	105 (50.5)	359 (54.7)	0.322
**Prior treatment failures**	4.9 ± 2.3	4.1 ± 2.2	5.2 ± 2.3	**< 0.001**
**BoNT/A responders**^*a*^	38 (10.3)	15 (23.1)	23 (7.5)	**< 0.001**
**≥** **1 comorbidity**	401 (46.4)	102 (49.0)	299 (45.5)	0.428
**Psychiatric comorbidities**	174 (20.5)	28 (13.9)	146 (22.6)	**0.007**
**HIT-6 score**	66.0 ± 9.2	65.1 ± 6.6	66.2 ± 9.9	0.133
**Erenumab**	639 (74.0)	169 (81.2)	470 (71.6)	
**Galcanezumab**	173 (20.0)	28 (13.5)	145 (22.1)	
**Fremanezumab**	52 (6.0)	11 (5.3)	41 (6.3)	
*Monthly regimen*	43 (5.0)	7 (3.4)	36 (5.5)	
*Quaterly regimen*	9 (1.0)	4 (1.9)	5 (0.8)	

*HFEM* High frequency episodic migraine, *CM* Chronic migraine, *BMI* Body mass index, *Underweight* < 18.5, *Normal weight* 18.5 to < 25, *Overweight* 25 to < 30, *Obesity* ≥ 30, *MMDs* Monthly migraine days, *MHDs* Monthly headache days, *MOH* Medication overuse headache, *NRS* Numerical Rating Scale, *UP* Unilateral pian, *UAs* Unilateral cranial autonomic symptoms, *BoNT/A* Onabotulinum toxin A, *HIT-6* Headache Impact Test-6. ^a^Proportion calculated on the 18 subjects who were treated with BoNT/A

The > 50%, > 75% and 100% response rates at week 24 were 64.9% (135/208), 30.8% (64/208) and 1% (2/208) in patients with HFEM, and 61.4% (403/656), 30.2% (198/656) and 2.4% (16/656) in patients affected by CM.

### Univariate analyses

In HFEM, we found a significant association between UP + UAs and both ≥50% response (61.8% vs 28%; *p* = 0.007) and ≥ 75% response (72.2% vs 43.1%; *p* = 0.005), and a trend for a positive association between UP + allodynia and ≥ 75% response rate (66.7% vs 47%; *p* = 0.056) (Tables 2 and 3).

**Table 2:** Demographic and clinical characteristics of < 50% responders and ≥ 50% responders in patients with high-frequency episodic (HFEM) or chronic migraine (CM)

	Number (%) or mean ± SD			Number (%) or mean ± SD		
	HFEM			CM		
	< 50% responders	≥50% responders	***p***-value	< 50% responders	≥50% responders	***p***-value
**Patients**	73 (35.1%)	135 (64.9%)		253 (38.6%)	403 (61.4%)	
**Age,** yrs	47.3 ± 10.2	48.6 ± 11.4	0.418	46.9 ± 11.9	48.3 ± 11.4	0.129
**Females**	58 (79.4)	100 (74.1)	0.386	204 (80.6)	313 (77.7)	0.366
**BMI**	226 ± 2.4	22.8 ± 2.9	0.549	23.8 ± 4.5	23.0 ± 3.5	**0.020**
**BMI class**			0.370			0.192
*Underweight*	4 (6.9)	4 (3.5)		12 (5.5)	22 (6.5)	
*Normal*	45 (77.6)	81 (71.1)		147 (67.4)	231 (67.7)	
*Overweight*	9 (15.5)	28 (24.5)		41 (18.8)	74 (21.7)	
*Obesity*	0	1 (0.9)		18 (8.3)	14 (4.1)	
**Disease duration*,*** yrs	29.6 ± 11.0	29.5 ± 13.0	0.971	29.5 ± 12.7	31.1 ± 12.7	0.094
**MMDs/MHDs at baseline**	10.7 ± 2.1	11.0 ± 2.0	0.439	24.3 ± 5.7	23.4 ± 5.8	**0.039**
**MOH**	–	–	–	220 (87.0)	351 (87.1)	0.958
**MOH duration,** yrs	–	–	–	9.3 ± 10.4	8.9 ± 7.9	0.593
**Monthly analgesic intake at baseline**	12.2 ± 5.2	13.0 ± 6.7	0.479	28.5 ± 23.0	26.7 ± 22.9	0.324
**NRS score**	7.7 ± 1.3	7.4 ± 1.5	0.206	7.8 ± 1.3	7.8 ± 1.2	0.997
**UP**	41 (56.9)	76 (58.9)	0.786	104 (42.4)	197 (49.5)	0.082
**Pain quality**			0.562			0.948
*Pulsating*	46 (65.7)	81 (64.3)		162 (67.2)	267 (68.5)	
*Pressing/tightening*	22 (31.4)	37 (29.4)		72 (29.2)	112 (28.7)	
*Other*	2 (2.9)	8 (6.3)		7 (2.9)	11 (2.8)	
**UAs**	27 (38.0)	53 (40.8)	0.704	115 (47.5)	211 (53.8)	0.123
**Allodynia**	28 (39.4)	61 (46.9)	0.307	143 (58.6)	240 (60.8)	0.589
**Dopaminergic symptoms**	46 (64.8)	100 (76.9)	0.065	167 (69.0)	250 (63.9)	0.191
**UP + allodynia**	11 (42.3)	44 (57.9)	0.169	59 (50.4)	147 (63.6)	**0.024**
**UP + UAs**	7 (28.0)	47 (61.8)	**0.007**	55 (47.0)	139 (60.4)	**0.017**
**UP + UAs + allodynia**	9 (52.9)	38 (61.3)	0.534	45 (57.0)	129 (70.1)	**0.039**
**Triptan responders**	50 (70.4)	89 (70.0)	0.925	133 (57.6)	240 (64.2)	0.105
**Concomitant prophylaxis**	34 (51.5)	71 (55.9)	0.668	148 (61.4)	211 (53.6)	0.058
**Prior treatment failures**	4.0 ± 2.0	4.2 ± 2.3	0.537	5.0 ± 2.2	5.3 ± 2.5	0.132
**BoNT/A responders**^*a*^	6 (40.0)	9 (60.0)	0.792	10 (43.5)	13 (56.5)	0.851
**≥** **1 comorbidity**	31 (42.5)	71 (52.6)	0.211	115 (45.4)	194 (48.1)	0.762
**Psychiatric comorbidities**	13 (18.1)	15 (11.5)	0.199	60 (24.4)	86 (21.6)	0.403
**HIT-6 score**	66.0 ± 7.3	64.6 ± 6.3	0.197	66.5 ± 8.5	66.1 ± 10.7	0.587

*HFEM* High frequency episodic migraine, *CM* Chronic migraine, *BMI* Body mass index, *Underweight* < 18.5, *Normal weight* 18.5 to < 25, *Overweight* 25 to < 30, *Obesity* ≥ 30, *MMDs* Monthly migraine days, *MHDs* Monthly headache days, *MOH* Medication overuse headache, *NRS* Numerical Rating Scale, *UP* Unilateral pain, *UAs* Unilateral cranial autonomic symptoms, *BoNT/A* Onabotulinum toxin A, *HIT-6* Headache Impact Test-6. ^a^Proportion calculated on the 18 subjects who were treated with BoNT/A

**Table 3:** Demographic and clinical characteristics of < 75% responders and ≥ 75% responders in patients with high-frequency episodic (HFEM) or chronic migraine (CM)

	Number (%) or mean ± SD			Number (%) or mean ± SD		
	HFEM			CM		
	< 75% responders	≥75% responders	***p***-value	< 75% responders	≥75% responders	***p***-value
**Patients**	144 (69.2%)	64 (30.8%)		458 (69.8%)	198 (30.2%)	
**Age,** yrs	47.5 ± 11.2	49.8 ± 10.5	0.167	47.6 ± 11.7	47.9 ± 11.5	0.815
**Females**	133 (78.4)	45 (70.3)	0.204	367 (80.1)	150 (75.8)	0.208
**BMI**	22.8 ± 2.5	22.6 ± 3.1	0.789	23.5 ± 4.1	23.0 ± 3.7	0.173
**BMI class**			0.457			0.848
*Underweight*	5 (4.3)	3 (5.5)		24 (6.1)	10 (6.0)	
*Normal*	88 (75.2)	38 (69.1)		261 (66.6)	117 (70.1)	
*Overweight*	24 (20.5)	13 (23.6)		83 (21.2)	32 (19.2)	
*Obesity*	0	1 (1.8)		24 (6.1)	8 (4.8)	
**Disease duration*,*** yrs	29.5 ± 11.9	29.6 ± 13.1	0.932	30.2 ± 12.8	31.3 ± 12.6	0.321
**MMDs/MHDs at baseline**	10.8 ± 2.1	11.0 ± 1.8	0.713	24.0 ± 5.8	23.3 ± 5.7	0.180
**MOH**	–	–	–	400 (70.0)	171 (30.0)	0.733
**MOH duration**, yrs	–	–	–	9.5 ± 9.8	8.0 ± 6.3	0.073
**Monthly analgesic intake at baseline**	12.6 ± 6.0	12.7 ± 4.1	0.926	28.0 ± 24.6	26.1 ± 18.6	0.333
**NRS score**	7.5 ± 1.5	7.6 ± 1.4	0.751	7.8 ± 1.3	7.7 ± 1.2	0.314
**UP**	77 (55.8)	40 (63.5)	0.305	201 (44.9)	100 (51.3)	0.134
**Pain quality**			0.770			0.951
*Pulsating*	87 (64.5)	40 (65.6)		301 (68.1)	128 (67.7)	
*Pressing/tightening*	42 (31.1)	17 (27.8)		129 (29.2)	55 (29.1)	
*Other*	6 (4.4)	4 (6.6)		12 (2.7)	6 (3.2)	
**UAs**	55 (39.9)	25 (39.7)	0.982	219 (49.3)	107 (56.3)	0.107
**Allodynia**	57 (41.3)	32 (50.8)	0.209	262 (58.6)	121 (63.0)	0.297
**Dopaminergic symptoms**	95 (68.8)	51 (81.0)	0.074	297 (669)	120 (63.5)	0.409
**UP + allodynia**	31 (47.0)	24 (66.7)	0.056	118 (53.6)	88 (68.8)	**0.006**
**UP + UAs**	28 (43.1)	26 (72.2)	**0.005**	111 (50.7)	83 (64.8)	**0.010**
**UP + UAs + allodynia**	26 (55.3)	21 (65.6)	0.360	96 (60.4)	78 (75.0)	**0.014**
**Triptan responders**	93 (68.9)	46 (7.0)	0.554	252 (59.4)	121 (66.8)	0.085
**Concomitant prophylaxis**	72 (54.5)	33 (54.1)	0.835	259 (58.6)	100 (51.8)	0.112
**Prior treatment failures**	5.6 ± 3.2	5.2 ± 3.0	0.471	6.9 ± 3.4	7.0 ± 3.4	0.777
**BoNT/A responders**^*a*^	9 (18.3)	6 (37.5)	0.216	19 (8.7)	4 (4.7)	0.350
**≥** **1 comorbidity**	74 (51.4)	28 (43.7)	0.386	213 (46.5)	86 (43.4)	0.522
**Psychiatric comorbidities**	19 (13.7)	9 (14.3)	1.000	106 (23.6)	40 (20.4)	0.372
**HIT-6 score**	64.9 ± 7.1	65.4 ± 5.4	0.657	66.3 ± 9.4	66.0 ± 11.0	0.754

*HFEM* High frequency episodic migraine, *CM* Chronic migraine, *BMI* Body mass index, *Underweight* < 18.5, *Normal weight* 18.5 to < 25, *Overweight* 25 to < 30, *Obesity* ≥ 30, *MMDs* Monthly migraine days, *MHDs* Monthly headache days, *MOH* Medication overuse headache, *NRS* Numerical Rating Scale, *UP* Unilateral pain, *UAs* Unilateral cranial autonomic symptoms, *BoNT/A* Onabotulinum toxin A, *HIT-6* Headache Impact Test-6. ^a^Proportion calculated on the 18 subjects who were treated with BoNT/A

In CM, ≥50% response rate was associated with lower BMI (23.0 ± 3.5 vs 23.8 ± 4.5; *p* = 0.020), lower MHD at baseline (23.4 ± 5.8 vs 24.3 ± 5.7; *p* = 0.039), UP + UAs (60.4% vs 47%; *p* = 0.017), UP + allodynia (63.6% vs 50.4%; *p* = 0.024), UP + UAs + allodynia (70.1% vs 57%; *p* = 0.039). The ≥75% response was associated with UP + UAs (64.8% vs 50.7%; *p* = 0.010), UP + allodynia (68.8% vs 53.6%; *p* = 0.006) and UP + UAs + allodynia (75% vs 60.4%; *p* = 0.014) (Tables 2 and 3). None of these results remained significant after correction for multiple comparison. No predictor of 100% response emerged in patients with HFEM or CM. 100% responders were on average older, had longer disease duration and lower analgesic intake at baseline (data not shown).

### Multivariate analysis

The logistic regression analysis showed that in HFEM both ≥50% and ≥ 75% responses were independently and positively associated with presence of UP + UAs (≥50% response OR: 4.23, 95%CI: 1.57–11.4; *p* = 0.004) (≥75% response OR: 3.44, 95%CI: 1.42–8.31; *p* = 0.006) (Table 4).

**Table 4:** Variables predicting ≥ 50% response and ≥ 75% response in patients with high frequency episodic migraine (HFEM): A logistic regression model

	**≥** 50% response rate		**≥** 75% response rate	
Variable	Odds ratio (95% CI)	*p*-value	Odds ratio (95% CI)	*p*-value
Sex				
M	1.00		1.00	
F	0.70 (0.34–1.44)	0.334	0.64 (0.32–1.27)	0.205
Age (yrs)	1.01 (0.98–1.04)	0.454	1.02 (0.99–1.05)	0.310
UP				
No	1.00		1.00	
Yes	1.11 (0.61–2.00)	0.738	1.43 (0.77–2.67)	0.262
UAs				
No	1.00		1.00	
Yes	1.17 (0.64–2.13)	0.614	0.99 (0.54–1.86)	0.995
UP + UAs				
No	1.00		1.00	
Yes	**4.23** (1.57–11.4)	**0.004**	**3.44** (1.42–8.31)	**0.006**

*UP* Unilateral pain, *UAs* Unilateral cranial autonomic symptoms, Hosmer–Lemeshow test for different models ranged from χ2 = 1.800 to χ2 = 8.960, with corresponding p-values ranging from 0.987 to 0.346

In CM, we found that ≥50% response was independently positively associated with UP (OR: 1.46, 95%CI: 1.02–2.08; *p* = 0.039), UAs (OR: 1.49, 95%CI: 1.05–2.11; *p* = 0.026), UP + UAs (OR: 1.90, 95%CI: 1.15–3.16; *p* = 0.012), UP + allodynia (OR: 1.71, 95%CI: 1.04–2.83; *p* = 0.034), and negatively associated with obesity (OR: 0.21, 95%CI: 0.07–0.64; *p* = 0.006). Conversely, ≥75% response was independently positively associated with and UP + UAs (OR: 1.78, 95%CI: 1.14–2.80; p = 0.012) (Table 5) and UP + allodynia (OR: 1.92, 95%CI: 1.22–3.06; *p* = 0.005). The models with UP + UAs combined were significantly better than those with UP and UAs separated, both for HFEM (AIC_50%_ = 108.7 vs 264.1 and AIC_75%_ = 130.6 vs 252.9) and for CM (AIC_50%_ = 370.9 vs 739.2 and AIC_75%_ = 457.8 vs 773.8). The combination of UP and UAs for HFEM patients significantly increased ≥ 50% response rate even after correction for multiple comparison, while the same combination is only borderline significant for ≥ 75% response rate. In the group of CM patients, the ORs for obesity and for the combination of UP and allodynia resulted borderline significant.

**Table 5:** Variables predicting ≥ 50% response and ≥ 75% response in patients with chronic migraine (CM): A logistic regression model

Variable	**≥** 50% response rate		**≥** 75% response rate	
	Odds ratio (95% CI)	*p*-value	Odds ratio (95% CI)	*p*-value
Sex				
M	1.00		1.00	
F	0.93 (0.60–1.35)	0.623	0.86 (0.57–1.30)	0.469
Age (yrs)	1.01 (0.99–1.02)	0.271	1.00 (0.99–1.02)	0.805
BMI				
Normal	1.00			
Underweight	1.19 (0.41–3.40)	0.750		
Overweight	1.06 (0.56–2.03)	0.849		
Obesity	0.21 (0.07–0.64)	**0.006**		
UP				
No	1.00		1.00	
Yes	1.35 (0.95–1.91)	0.093	1.29 (0.92–1.81)	0.133
UAs				
No	1.00		1.00	
Yes	1.49 (1.05–2.11)	**0.026**	1.34 (0.95–1.88)	0.099
Allodynia				
No	1.00		1.00	
Yes	1.14 (0.79–1.64)	0.483	1.23 (0.86–1.74)	0.253
UP + allodynia				
No	1.00		1.00	
Yes	1.71 (1.04–2.83)	**0.034**	1.92 (1.22–3.06)	**0.005**
UP + UAs				
No	1.00		1.00	
Yes	1.90 (1.15–3.16)	**0.012**	1.78 (1.14–2.80)	**0.012**

*UP* Unilateral pain, *UAs* Unilateral cranial autonomic symptoms, *BMI* Body mass index, *Underweight* < 18.5, *Normal weight* 18.5 to < 25, *Overweight* 25 to < 30, *Obesity* ≥ 30, Hosmer–Lemeshow test for different models ranged from χ2 = 1.233 to χ2 = 8.420, with corresponding *p*-values ranging from 0.996 to 0.394

## Discussion

New costly targeted treatments prompt to reconsider migraine management in terms of customized healthcare and tailored therapy in the modern precision medicine era [27]. Clinical predictors could favor personalized therapy in migraine, because despite the advances in the understanding of its pathophysiology no reliable disease biomarker exists to date [28, 29].

**Fig. 1:**
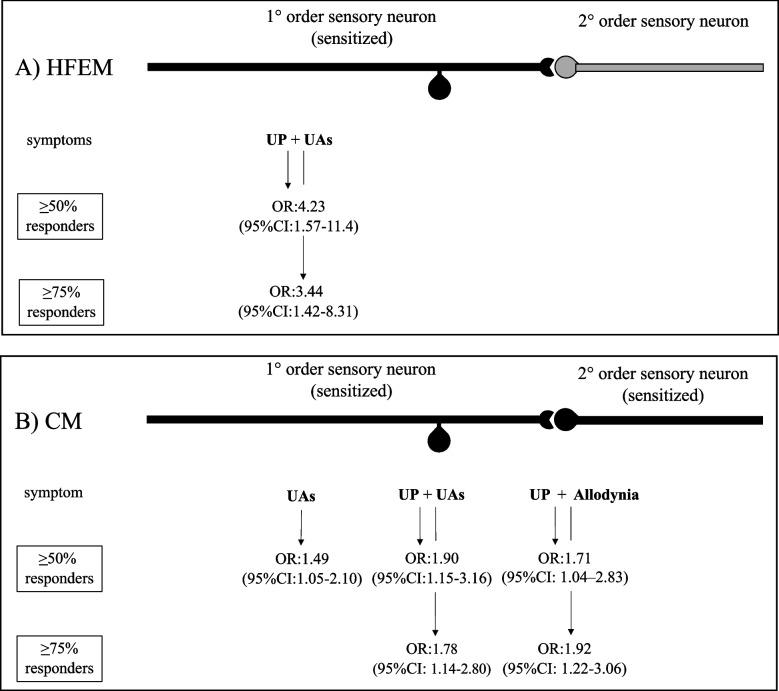
**A** In patients with HFEM, unilateral pain (UP) associated with unilateral cranial autonomic symptoms (UAs) – clinical manifestation of CGRP-mediated peripheral sensitization - predict both ≥ 50 and ≥ 75% response to antiCGRP mAbs. **B** In patients with CM, UAs and UP + UAs predict ≥ 50 response to antiCGRP mAbs, while UP + UAs and UP associated with allodynia - a CGRP-mediated symptom of central sensitization - predict ≥ 75% response

The main finding of the present study is that easily obtainable clinical features could be of help in predicting response to antiCGRP mAbs. In fact, we document that the most reliable predictor of ≥50% and ≥ 75% responses to antiCGRP mAbs in HFEM is a combination of symptoms related to trigeminal sensitization (UP + UAs), while in CM is a combination of symptoms referred to both peripheral sensitization and central sensitization (allodynia) (Fig. 1). These observations echo the hypothesis raised by Hargreaves and Olesen who astutely questioned whether CGRP hyperresponders could have *“exaggerated sensory (allodynia) or autonomic signs such as flushing or vasodilation in tissues innervated by the trigeminal system during their attacks suggestive of sensory activation*” [30]. This pooling of different study outcomes, as planned in advance, increases statistical precision due to the higher event rates, and allows to avoid competing risks in outcome assessment when there is no obvious choice of a primary trial outcome. In addition, this approach helps investigators to avoid an arbitrary choice between several important outcomes that refer to the same disease process, and to have a deeper insight into pathogenetic mechanisms.

Our results fit well with current knowledge on the role of CGRP in the genesis of migraine and of its chronification [31]. During the migraine attack, CGRP is antidromically released from peripheral nociceptive endings, and triggers a cascade of events ultimately leading to peripheral trigeminal sensitization [32]. Indeed, we found that HFEM responders to antiCGRP mAbs have symptoms of intense CGRP-related trigeminal activation, being characterized by a unilateral headache (UP) tracing the overactive primary afferent sensory neurons accompanied by homolateral cranial parasympathetic symptoms (UAs) due to the activation of the trigemino-autonomic reflex, a physiological defensive response to intense trigeminal stimuli [33]. CGRP also contributes to sensitize second-order nociceptive neurons within the central nervous system, favoring the development of central sensitization, the pathophysiological condition underpinning CM**.** Not surprisingly, the endophenotype of CM responder to antiCGRP mAbs is characterized by symptoms of peripheral sensitization (UP, UAs) coupled to allodynia, the clinical manifestation of central sensitization [33].

Obesity emerged as a negative predictor of antiCGRP mAbs responsiveness in patients with CM. A possible explanation is that although increased neuropeptides’ release in patients with trigeminal overactivation seems associated with a favorable response to trigeminal-targeted treatments, current antiCGRP mAbs treatments might be unable to properly counteract the excessive CGRP activity characterizing obese individual [29, 34, 35]. Weight reduction strategies could thus be advantageous in increasing antiCGRP mAbs responsivity in these patients.

The present study points out that pain characteristics are more relevant than other clinical or sociodemographic factors in determining antiCGRP mAbs response. Further, their accurate assessment may represent one way to envisage different pain-generating mechanisms [28]. The concept that precise pain profiling may be helpful in unravelling its distinct pathophysiological machinery and in improving treatment is well established in pain research [36]. The sodium channel blocker oxcarbazepine provided equivocal findings in peripheral neuropathic pain but showed indeed clear-cut different therapeutic effects when tested in a phenotype-stratified clinical trial differentiating patients with the irritable vs the non-irritable nociceptor sensory profile [37]. Thus, efforts are needed also in migraine to identify different mechanism-based endophenotypes which could aid its diagnosis and treatment [38]. In previous works, we documented that patients showing symptoms of trigeminal peripheral sensitization (UP + UAs) are likely to be more sensitive to triptans and, broadly speaking, to trigeminal-targeted treatments [39–41]. The present study extends this hypothesis also to antiCGRP mAbs. The relevance of pain characteristic in predicting therapeutic response in migraine has been pointed out also by other research groups. Sarchielli et al. documented that rizatriptan responders have clinical and biochemical evidence of increased trigeminal activation [42]. Directionality and site of pain have been considered neurological markers to single out botulinum toxin responders in migraine by Jakubowski et al., who reported considerable differences in the responders’ rates between patients with imploding, ocular o exploding headache (94%, 100% and 19%, respectively) in a migraine population including a large proportion (57.1%) of patients affected by the episodic form, usually considered unresponsive to onabotulinum toxin A [43]. Likewise, migraine patients with imploding or ocular headache are more likely to be super-responders (> 75% reduction in monthly headache days) to rimabotulinumtoxin B compared to those with exploding pain. For the above reasons, it has been suggested to include subjective pain perception in migraine diagnosis [44].

This study has several limitations. Firstly, the proportion of patients treated with the diverse antiCGRP mAbs is heterogeneous and not comparable (erenumab 74%; galcanezumab: 20%; fremanezumab 6%). This discrepancy reflects the different pre-reimbursement access to the various antiCGRP mAbs in our Country, erenumab having been available since December 2018, galcanezumab since September 2019 and fremanezumab since July 2020. Secondly, our study does not include eptinezumab, not yet approved in Italy. Thirdly, among patients affected by episodic migraine, we considered only those having at least 8 monthly migraine day (according to Italian reimbursement rules) and therefore our findings cannot be simply transferred to patients affected by lower frequency episodic migraine. Lastly, we acknowledge that the factors investigated as potential predictors could sound somehow arbitrary and, in any case, do not exclude the existence other predictive characteristics. The main strength of this study is surely the large number of patients recruited by several headache centers nationwide and interviewed - after method standardization - with a shared semi-structured questionnaire to obtain comprehensive information on sociodemographic and clinical features.

In conclusion, our study suggests that a critical evaluation of easily obtainable patient-reported clinical findings - such as migraine pain characteristics indicating peripheral or central sensitization - may be of help in predicting responsiveness to antiCGRP mAbs in HFEM and CM. In addition, a more precise pain profiling may represent a steppingstone for a mechanism-based approach and personalized treatment of migraine with compounds targeting specific molecular mechanisms. Future drug trials should hopefully provide a better definition of migraine phenotype to minimize migraine pathophysiological heterogeneity and to favor tailored therapy to the individual patient [28].

## Appendix

**Table 6:** Coinvestigators

Name	Location	Role	Contribution
Laura Di Clemente	San Camillo Hospital, Rome	S.I.	Collection and evaluation data
Paola Di Fiore	ASST Santi Paolo e Carlo, Milan	S.I.	Collection and evaluation data
Nicoletta Brunelli	Campus Bio-Medico, Rome	S.I.	Collection and evaluation data
Maria *C. costa*	Campus Bio-Medico, Rome	S.I.	Collection and evaluation data
Bruno Colombo	San Raffaele Scientific Institute, Milan	S.I.	Collection and evaluation data
Ilaria Cetta	San Raffaele Scientific Institute, Milan	S.I.	Collection and evaluation data
Luigi d'Onofrio	Campus Bio-Medico, Rome	S.I.	Collection and evaluation data
Gerardo Casucci	Casa di Cura S. Fracesco, Benevento	S.I.	Collection and evaluation data
Domenica Le Pera	IRCCS San Raffaele Roma, Rome	S.I.	Collection and evaluation data
Carlo Tomino	IRCCS San Raffaele Roma, Rome	S.I.	Led and coordinated communication among sites
Giovanna Viticchi	Politechnic Universuty, Ancona	S.I.	Collection and evaluation data
Antonio Salerno	S. Giovanni Addolorata Hospital, Rome	S.I.	Collection and evaluation data
Bruno Mercuri	S. Giovanni Addolorata Hospital, Rome	S.I.	Collection and evaluation data
Licia Grazzi	IRCCS Carlo Besta, Milan	S.I.	Collection and evaluation data
Domenico D’Amico	IRCCS Carlo Besta, Milan	S.I.	Collection and evaluation data
Cecilia Camarda	University of Palermo	S.I.	Collection and evaluation data
Massimo Autunno	University of Messina	S.I.	Collection and evaluation data
Alessandro Valenza	Belcolle Hospital, Viterbo	S.I.	Collection and evaluation data
Steno Rinalduzzi	S. Camillo de Lellis Hospital, Rieti	S.I.	Collection and evaluation data
Miriam Tasillo	S. Camillo de Lellis Hospital, Rieti	S.I.	Collection and evaluation data
Giuliano Sette	Sant’Andrea University Hospital, Rome	S.I.	Collection and evaluation data
Giorgio Spano	AOU Mater Domini Hospital, Catanzaro	S.I.	Collection and evaluation data

*S.I*. Site investigator
